# Measuring anticipated stigma towards irritable bowel syndrome (IBS) in the German general population: testing the applicability of a modified version of the Perceived Stigma Scale of IBS in the cross-sectional SOMA.SOC study

**DOI:** 10.1136/bmjopen-2024-097149

**Published:** 2025-11-04

**Authors:** Anna Christin Makowski, Rieke Barbek, Anne Toussaint, Bernd Löwe, Olaf von dem Knesebeck

**Affiliations:** 1 Department of Medical Sociology, University Medical Center Hamburg-Eppendorf, Hamburg, Germany; 2 Department of Psychosomatic Medicine and Psychotherapy, University Medical Center Hamburg-Eppendorf, Hamburg, Germany

**Keywords:** Cross-Sectional Studies, Irritable Bowel Syndrome, Psychometrics

## Abstract

**Abstract:**

**Objective:**

There is only a little research on anticipated stigma in the general population, despite evidence of negative consequences with regard to underutilisation of medical testing or treatment. While a lot of instruments focus on the interpersonal dimension of public stigma (i.e., societal attitudes), fewer assess the intrapersonal dimension of anticipated stigma, a belief that stigmatising attitudes will be directed at the self in the future. The objective of this study was to test the applicability and the psychometric properties of an anticipated stigma scale in a population survey on beliefs about irritable bowel syndrome (IBS).

**Methods:**

Analyses are based on telephone interviews in a random population sample of 1205 adult individuals in Germany. They were presented with a vignette describing a person with symptoms suggestive of IBS, followed by 10 items assessing anticipated stigma based on a modified version of the Perceived Stigma Scale of IBS.

**Results:**

Results indicate that individuals expected others not to have enough knowledge about symptoms and may ascribe their aetiology to personal behaviour. A first exploratory factor analysis (EFA) yielded two factors. Examination of scree plot and content considerations justified a second EFA specifying a one-factorial solution with Cronbach’s α of 0.80 and satisfactory discriminatory power and mean inter-item correlations.

**Conclusion:**

The applicability of the scale to assess anticipated IBS stigma in the general population using a vignette design was demonstrated. Such assessments can be used as the basis for tailored anti-stigma measures, for example, the communication of specific facts about the development of IBS symptoms.

**STRENGTHS AND LIMITATIONS OF THIS STUDY**

- We report acceptable psychometric properties of a modified Perceived Stigma Scale for irritable bowel syndrome (IBS) in a large random sample of the German general population.
- Case vignettes developed by clinicians and specialists allowed for the presentation of the complex medical condition of IBS in a standardised way.
- A non-response rate of 55% raises the possibility of selection bias.
- The assessment of anticipated stigma among the general public was primarily conducted with participants who do not have IBS, requiring them to abstract from a vignette instead of relying on their own illness experiences.

## Introduction

 Stigma is a pervasive social phenomenon that has increasingly become a focus of attention due to its profound effects on individuals.^1^ It encompasses multiple dimensions, each contributing to its complex nature. On an interpersonal level, public stigma refers to societal attitudes and beliefs towards individuals with a particular condition, often leading to discrimination and social exclusion.^2^ The intrapersonal dimension of stigma can be captured by perceived, anticipated and internalised (also called self-) stigma.^3^ Self- or internalised stigma is the extent to which people endorse negative beliefs and feelings associated with their stigmatised attribute and apply them to the self.^4^ Perceived stigma involves individuals’' perceptions of how they are viewed by others and is the extent to which people perceive stereotyping, prejudice and discrimination directed at them from others.^5^ Anticipated stigma differs from this in that it has not been directly experienced or perceived. It can be described by the worry that prejudice, discrimination and stereotyping will be directed at the self from others in the future.^6^ Accordingly, anticipated stigma can also be present in the general population and reflects hypothetical views on how individuals with a potentially stigmatised condition may be assessed and treated.

Anticipating negative consequences from others in case of being personally affected by or disclosing an illness can have far-reaching implications for the individual. Anticipated stigma has been found to be a predictor of psychological distress as well as underutilisation or avoidance of needed healthcare services among people with concealable stigmatised identities, i.e., history of mental illness, domestic violence or epilepsy, for example.^7^ For individuals living with chronic illness, anticipated stigma from family and friends or within healthcare settings was associated with poorer routine and emergent healthcare utilisation as well as reduced quality of life.^5 6 8^ Further study results indicated that greater anticipated stigma was a predictor of poorer medication adherence^9^ and fewer referrals to needed mental-health care among older adults with depressive symptoms.^10^ These negative consequences of anticipated stigma highlight the critical importance of measuring this phenomenon. The importance of assessing anticipated stigma also at population level is underlined by the label avoidance theory, according to which people who anticipate stigma engage less in medical testing, screening or examinations in order to avoid the label of a stigmatising disease.^11^ Research has shown this in samples of the general population for COVID-19 and HIV.^12 13^

Fox and colleagues^14^ critically reviewed 400 survey instruments to assess stigma. They found that most dealt with public stereotypes and discrimination, while the intrapersonal dimension (experienced, anticipated or self-stigma) received less attention. Instruments that measure anticipated stigma often focus on the perspective of those affected, whereas the beliefs at population level are overlooked. However, in contrast to other dimensions of stigma, anticipated stigma can be measured from two different perspectives. Those affected can assess anticipated stigmatisation from their point of view, but those not (yet) affected can also empathise with situations and anticipate reactions. Quinn and Chaudoir,^7^ for example, developed an anticipated stigma scale that was composed of nine items of the ‘day-to-day’ discrimination scale,^15^ which measures the frequency and nature of everyday discriminatory experiences that individuals might face, plus six items that focused more on relationship devaluation. Another example is the Chronic Illness Anticipated Stigma Scale (CIASS) by Earnshaw *et al.*,^16^ a 12-item scale with three subscales measuring anticipated stigma from friends and family, colleagues and healthcare workers. The review mentioned above concluded that instruments that capture anticipated stigma and that can also be used in public surveys are least common.^14^ When eliciting public beliefs and attitudes, stigma research often makes use of vignettes.^17^,^19^ By presenting respondents with hypothetical scenarios or stories (vignettes), they are prompted to reflect on how they would think or act in similar real-life circumstances. Vignettes can provide context to complex issues and are an engaging way to present a scenario, leading to more authentic responses. This seems highly suitable for a complex medical condition such as irritable bowel syndrome (IBS). Interviewees are given the opportunity to better empathise with the situation so that they can then be asked about anticipated stigma.

The present research focused on symptoms associated with IBS. IBS is a functional disorder that is caused by a multifactorial pathophysiology and characterised by chronic abdominal pain or discomfort and alteration of bowel habits.^20^ Due to historically assumed psychogenic causes and the socially undesirable topic of bowel functioning, IBS is associated with stigma, which poses further unique challenges on those affected. In their review, Hearn *et al.*^21^ gave an overview of studies on how patients with IBS face disbelief and trivialisation of their symptoms and experience feelings of isolation, frustration and shame. It is known that stigma-related barriers may impede treatment adherence and exacerbate symptom severity in IBS, aggravating a cycle of distress and avoidance behaviour.^22^ In order to gain a deeper insight into the experience of the patients living with IBS and to identify sources of stigmatisation, Jones and colleagues^23^ have developed the perceived stigma scale of IBS (PSS IBS). A multi-stage process involving those affected resulted in 10 items assessing perceived stigma in different social settings, for example, family and friends, the workplace and healthcare. The scale focused exclusively on patients’ experiences; the instrument and its phrasing did not initially take into account assumptions made by the general population.

To our knowledge, there is no study that has addressed anticipated stigma in IBS. Moreover, there is a lack of instruments measuring anticipated stigma in the general population. Against this background, we propose an anticipated stigma scale that can be used in population surveys with reference to vignettes. While there are scales to measure anticipated stigma, we adapted the condition-specific scale developed by Jones *et al.*,^23^ originally designed for IBS. Our rationale was that the IBS context is unique, so starting from an IBS-specific instrument was preferable. The aforementioned instruments^7 16^ were broader, but not ideal for vignette-based population survey research focused on IBS. For this purpose, we used a modified version of the PSS IBS. This study aimed to test the modified scale with regard to its applicability and psychometric properties.

## Method

### Study design and sample

The data originate from a national telephone survey (CATI, computer-assisted telephone interview) among the adult population (18 years or older) conducted in Germany between March and May 2022 by a company specialised in social research. Participants were randomly selected, applying a dual-frame approach with random-digit-dialling to include 70% registered and computer-generated telephone numbers and 30% mobile numbers. A combination of these options was considered a methodological necessity, especially due to groups that are rather difficult to reach and so-called ‘mobile-only’ users. For mobile numbers, target persons were owners or main users of the mobile phone. The connection was considered a neutral drop-out if the respondent was younger than 18 years. In households contacted via landline, the Kish-Selection-Grid^24^ was applied to randomly choose a participant. Oral informed consent was given in the beginning of the interview.

There were 2413 completed interviews available. In total, 5397 (net sample) persons had been contacted. Of these, around 19% could not be reached, while 36% refused to participate, resulting in a response rate of about 45%. The sample comprised 49.5% female respondents, with an average age of 52 years (SD=18.5). Around 37% had a high level of education (≥ 12 years). For more details, please also see the publication by von dem Knesebeck *et al*.^25^ At the beginning of the interviews, a vignette describing a person with typical symptoms of IBS or fatigue was presented to the respondents (for details please see below and online supplemental material). One of the two vignettes (IBS or fatigue) was randomly assigned to half of the sample, respectively. As the following analyses will focus on IBS, half of the total sample (n = 1205) will be used.

The study was conducted in the framework of a project on social inequalities in aggravating factors of persistent somatic symptoms (SOMA.SOC), which is embedded in the Research Unit 5211 ‘Persistent SOMAtic Symptoms ACROSS Diseases: From Risk Factors to Modification (SOMACROSS)’.^26^ The overarching aim of the study was to investigate social inequalities in factors that contribute to symptom persistence in IBS and fatigue. The cross-sectional survey of the general population included questions on possible aggravating factors of persistent symptoms, for example perception of illness, health literacy, stigma and illness and treatment experiences. In the present study, we solely focus on IBS-related data. The methodology of SOMA.SOC is described in detail in the study protocol.^27^

### Vignettes

In the beginning of the survey, a pre-recorded vignette was presented to the respondents that conveyed signs and symptoms indicative of IBS. Following the vignette, participants were asked about, for example, their knowledge and experiences in relation to the symptoms described, including questions about anticipated stigma. The vignette was developed in close cooperation with specialists from the fields of psychosomatics, gastroenterology, general and internal medicine and was based on the International Classification of Diseases 10 criteria.^28^ While the disease-specific symptoms (eg, abdominal cramps and diarrhoea (please see online supplemental appendix for details)) remained unchanged, the vignette varied with regard to gender (male/female), migration history (yes/no) and occupational status (lawyer/cleaner) of the person depicted. This led to eight IBS vignettes that were each randomly assigned to around 150 respondents. For the present analyses, these variants of the IBS vignette were pooled. We used unlabeled vignettes, i.e., participants were not informed that the person in the vignette has IBS. In order to neutralise possible interviewer effects and increase reliability, the vignettes were audio-recorded by a trained speaker and directly played to the respondents from the computer.

### Instrument

#### The original scale

For this study, we made use of a modified version of the PSS IBS, an instrument developed by Jones *et al*^23^ to measure perceived stigma across six relevant social domains (family, friends, healthcare provider, significant other, coworkers, employer). The original instrument was applied among IBS patients and assesses the frequency (0=‘never’ to 5=‘always’) individuals have experienced common stigmatising issues (10 items), such as symptoms not being taken seriously, people not having enough knowledge about symptoms or keeping IBS symptoms hidden out of fear of being treated differently. For each of the six domains, the participants were asked to rate the frequency of the 10 stigmatising experiences. The score for each of the six sub-scales was computed as a mean score (ranging from 0 to 5); furthermore, a global stigma score was computed as the sum of the 10 items across all domains ranging from 0 to 50. Thus, the PSS IBS can be scored twofold: across items for a global stigma score and across social domains to identify interpersonal areas associated with greater perceived stigma. In the study of Jones *et al*, measures of reliability and validity were excellent, Cronbach’s α was estimated at 0.91, mean inter-item correlation was 0.50 and ranged from 0.29 to 0.71. However, detailed results of factor analyses were not reported.^23^

#### Adaptations for the present study

Whereas the PSS IBS measures perceived stigma, our study used vignettes and surveyed the general population, a methodological approach specifically designed to measure anticipated stigma in individuals both with and without lived experience of the condition, as this type of stigma reflects hypothetical views and is not contingent on direct personal experience. We made several key adaptations to the PSS IBS for this purpose. Although it was assumed that there were people in the sample who suffered from IBS-like symptoms, the majority (64.6%) were not affected.^25^ Moreover, in the total sample, the stigma scale was not only used for the IBS vignette but also for the fatigue vignette. With this in mind, and given the intention to propose an instrument that can be used for different conditions, we made the following modifications:

##### Wording

The wording of the items has been adapted and formulated in conjunctive mode so that the scale can be used generically. The individual items now only referred to ‘symptoms’, but not to ‘IBS symptoms’.

##### Social domains

Due to the different focus of this survey, as well as time restrictions in the telephone survey, it was decided not to ask about anticipated stigma in the six different domains. The 10 items were introduced with the following question: “To what extent do you think the following statements apply if you yourself were affected by complaints like those of Mrs./Mr. E.?”.

##### Translation

As no German-language version was previously available that was also suitable for use in the general population, the original items of the scale were translated and adapted independently by members of the research group (ACM, RB). In addition, a translation and back-translation were carried out with the support of a native speaker. These translations were then discussed and agreed internally.

##### Response scale

To suit the context of our study, we modified the original scale’s response options. This design decision facilitated clear communication during the telephone interviews, reduced respondent burden and avoided a tendency to the middle. A fully verbalised four-point response scale was used (‘completely disagree’, ‘rather disagree’, ‘rather agree’, ‘completely agree’), thus measuring the degree of agreement with the individual items. Respondents who could not or did not want to commit themselves when answering the questions were given the option of selecting a ‘don’t know’/‘no answer’ category (please see table 1 for description of items). This makes the response metric ordinal rather than continuous. To address potential consequences of this choice, we also present all descriptive statistics appropriate for ordinal items (item medians and interquartile ranges) and performed sensitivity analyses using factor extraction methods appropriate for ordinal data (see Statistical analyses)

**Table 1:** Response frequencies (%) including missing values (‘don’t know’/‘no answer’), n=1205

To what extent do you think the following statements apply if you yourself were affected by symptoms like those of Mr./Mrs. E.?	Completely disagree	Rather disagree	Rather agree	Completely agree	Missing values
My symptoms would not be taken seriously by other people.	16.8	35.7	35.4	10.5	1.7
My symptoms would be believed to be more ‘in my head’ than physical.	16.1	34.6	36.8	10.2	2.3
I would keep my symptoms hidden from other people because they would treat me differently.	27.6	37.3	25.9	8.2	1.0
My symptoms would be believed to be caused by something I’m doing or have done.	12.4	30.3	43.9	8.7	4.7
I would not feel I could be as open about my symptoms as I’d like to be.	18.9	31.4	36.1	11.8	1.8
Some people would not have enough knowledge about my symptoms.	7.4	25.1	45.4	18.6	3.5
Some people would not be interested in hearing about my symptoms.	13.0	34.3	38.3	12.9	1.5
If some people knew about my symptoms, they would treat me differently.	14.9	37.2	37.7	7.6	2.6
Some people would not understand if I had to make changes to plans because of my symptoms.	13.7	39.3	34.7	9.6	2.7
I would worry that some people would pass me over or limit my opportunities if they knew I had these symptoms.	22.7	39.5	27.6	8.5	1.7

##### Scoring

For further analyses, we inverted the items so that higher agreement corresponds to greater anticipated stigma. Therefore, values from 0 ‘completely disagree’ to 3 ‘completely agree’ were assigned. On this basis, a stigma sum scale was calculated ranging from 0 to 30. Incomplete data due to missing values were excluded.

### Statistical analyses

In descriptive analyses, frequencies and missing values were calculated for the 10 items. With regard to distribution characteristics, mean, SD, median and IQR were calculated for the items. Histograms and Q-Q plots were used to test for normal distribution in addition to skewness and kurtosis. We also looked at the single item discriminatory power, which should not be less than 0.3^29^ as well as the mean inter-item correlation, which should range between 0.15 and 0.50.^30^ We calculated the item difficulty by dividing the item mean by the maximum possible response category (ie, 3). Against the background of the changes made to the original version, the scale was explored using exploratory factor analysis (EFA). To this end, the maximum likelihood procedure with oblique rotation promax was carried out. To identify the number of factors, eigenvalue, Scree test and content-related considerations were taken into account. Factor loadings of 0.3 or higher were considered interpretable.^31^ To investigate the internal consistency, Cronbach’s α was determined, with α values of ≥0.7 being considered as satisfactory.^32^ These analyses were carried out using SPSS 29.^33^ In addition, given the ordinal nature of the item response, we performed sensitivity analyses. A polychoric correlation matrix was calculated using the psych package in R. EFA was then performed on this matrix using principal axis factoring to extract a single factor. These analyses were conducted using the fa.poly function from the psych package in R.^34^ For results of sensitivity analyses, please see online supplemental material.

## Results

Descriptive analysis of the 10 items is presented in table 1. The item “Some people would not have enough knowledge…” received the highest agreement with 64.0% (answer categories rather agree and completely agree combined), the highest disagreement was expressed for the item “I would keep my symptoms hidden…” (64.9%, answer categories rather disagree and completely disagree combined). Missing values ranged between 1.0% (“I would keep my symptoms hidden…”) and 4.7% (“My symptoms would be believed to be caused by something I'm doing…”). Further characteristics of the scale are presented in table 2. In terms of item difficulty, we found that the item “Some people would not have enough knowledge…” had the lowest difficulty (0.59), while the highest item difficulty value was achieved for the item “I would keep my symptoms hidden….” (0.38). Skewness and kurtosis indicated a non-normal distribution of the items (table 2). Histograms also contradicted a normal distribution (results not shown).

**Table 2:** Distribution characteristics of the anticipated stigma scale items (n=1148–1193)

To what extent do you think the following statements apply if you yourself were affected by symptoms like those of Mr./Mrs. E.?	Mean (SD)	Item difficulty	Median	IQR	Skewness	Kurtosis
My symptoms would not be taken seriously by other people.	1.40 (0.89)	0.47	1.00	1.00	0.025	−0.786
My symptoms would be believed to be more ‘in my head’ than physical.	1.42 (0.89)	0.47	1.00	1.00	−0.020	−0.751
I would keep my symptoms hidden from other people because they would treat me differently.	1.15 (0.92)	0.38	1.00	2.00	0.337	−0.786
My symptoms would be believed to be caused by something I’m doing or have done.	1.51 (0.83)	0.50	2.00	1.00	−0.244	−0.544
I would not feel I could be as open about my symptoms as I’d like to be.	1.42 (0.93)	0.47	1.00	1.00	−0.022	−0.899
Some people would not have enough knowledge about my symptoms.	1.78 (0.84)	0.59	2.00	1.00	−0.332	−0.437
Some people would not be interested in hearing about my symptoms.	1.52 (0.88)	0.51	2.00	1.00	−0.063	−0.705
If some people knew about my symptoms, they would treat me differently.	1.39 (0.84)	0.46	1.00	1.00	−0.034	−0.636
Some people would not understand if I had to make changes to plans because of my symptoms.	1.41 (0.85)	0.47	1.00	1.00	0.066	−0.614
I would worry that some people would pass me over or limit my opportunities if they knew I had these symptoms.	1.22 (0.90)	0.41	1.00	1.00	0.264	−0.727

Item scale 0 ‘completely disagree’ to 3 ‘completely agree’.

Based on the Kaiser-Meyer-Olkin (KMO) measure of sampling adequacy (MSA) and Bartlett’s test of sphericity, the assumptions for performing EFA were met (KMO: 0.86, Bartlett’s: <0.001). The MSA for single items ranged between 0.84 and 0.89. The initial EFA suggested a two-factorial solution (results not shown), whereby the two factors correlated with each other. However, the scree plot and a low eigenvalue of the second factor (1.022) justified a single factor solution. As a unidimensional construct was also considered appropriate in terms of content, another EFA with the specification of a single-factorial solution was computed, which explained 28.94% of the total variance. The results presented in table 3 show that all factor loadings exceeded 0.30. For a scale containing all 10 items, Cronbach’s α was 0.80 and could not be further increased by deleting individual items. The discriminatory power showed a satisfactory item-to-scale correlation (>0.30) for all 10 items. The stigma sum score was approximately normally distributed, with a mean of 14.54 (SD=5.46) and a median of 15.00. The data exhibited a skewness of −0.062 (SE=0.075) and a kurtosis of −0.121 (SE=0.149), with histograms and Q-Q plots further supporting a normal distribution. The mean inter-item correlation was 0.28 and ranged from 0.13 to 0.47 (results not shown).

**Table 3:** Explorative factor analysis, single factor solution (maximum likelihood; n=1051)

Item	Factor loadings	Discriminatory power	α if item deleted
My symptoms would not be taken seriously by other people.	0.528	0.468	0.782
My symptoms would be believed to be more ‘in my head’ than physical.	0.505	0.451	0.784
I would keep my symptoms hidden from other people because they would treat me differently.	0.600	0.525	0.775
My symptoms would be believed to be caused by something I’m doing or have done.	0.353	0.316	0.798
I would not feel I could be as open about my symptoms as I’d like to be.	0.666	0.581	0.768
Some people would not have enough knowledge about my symptoms.	0.519	0.470	0.782
Some people would not be interested in hearing about my symptoms.	0.548	0.477	0.781
If some people knew about my symptoms, they would treat me differently.	0.482	0.432	0.786
Some people would not understand if I had to make changes to plans because of my symptoms.	0.514	0.459	0.783
I would worry that some people would pass me over or limit my opportunities if they knew I had these symptoms.	0.605	0.532	0.774
Total variance explained	28.94%		
Overall Cronbach’s α			0.80

## Discussion

The aim of our study was to test the applicability and the psychometric properties of an anticipated stigma scale in a population survey on beliefs about IBS. To this end, we made use of a modified version of the PSS IBS developed by Jones *et al*.^23^

The single items of the adapted scale in German were not normally distributed, while a normal distribution could be assumed for the sum scale calculated. Similar to the original study by Jones *et al*,^23^ the items relating to low knowledge about symptoms and lack of interest achieved the highest approval rates. In contrast to the original, the anticipated stigma regarding an attribution of personal responsibility (“…caused by something I’m doing or have done.”) tended to elicit more approval from respondents in the German context. In the original version of the scale, there was no option of item non-response (‘don’t know/no answer’). In our survey, missing values varied between 1.0% and 4.7% and can therefore be considered minor. Nevertheless, it should be noted that there were respondents who preferred not to respond to a statement on one of the stigma items. On the one hand, this could mean that the required level of abstraction was too high. Looking at the item “…caused by something I’m doing…” in particular, the possible reasons for the occurrence of the symptoms described in the vignette may seem so varied to the respondents that they were too uncertain to make a concrete assessment. On the other hand, item non-response could perhaps also be interpreted in the context of social desirability, so as not to indicate something supposedly ‘wrong’ or ‘pejorative’. This is particularly relevant in stigma research, where respondents are asked to make assessments about people with certain characteristics, even if our survey solely concerns anticipated stigma.

The interpretation of the scree plot and content rationale justified the extraction of a single factor for anticipated stigma, as has been found in previous research.^35^ The one-factorial solution accounted for 29% of the total variance and the internal consistency of the model was satisfactory with α=0.80.^36^ There were two items with low factor loadings (<0.50), namely “…caused by something I’m doing…” and “If people knew, …they would treat me differently.” However, since we tended to rule out random loading patterns due to the sample size and the values can still be considered satisfactory,^31^ we decided to retain the items.

Comparisons with other (validation) studies of the PSS IBS are difficult, as these focused on perceived stigma among patients. Cronbach’s α coefficients in these studies were higher than in the present study and ranged between 0.87 and 0.92.^3537^,^39^ A possible explanation became apparent when looking at the mean inter-item correlations. The comparison showed that associations between the items in this study (0.28) were less pronounced than those in the original version (0.50). This divergence may be due to the different language or country, the different population (patients vs public) and the differing concepts of stigma (perceived vs anticipated). Furthermore, our assessment of anticipated stigma requires a high level of abstraction. In our study, the majority of respondents (64.6%) were not themselves affected by the symptoms described in the vignettes.^25^ It is also discussed that, in order to be able to anticipate stigma, it is essential to be at least aware of public or structural stigma.^14^ This awareness may vary among the respondents. However, as the values regarding inter-item correlations were within an acceptable range, deviations can be tolerated.^30^

Given the range and overall mean (14.54) of the sum scale, anticipated stigma in the present sample was rather moderate. In the study by Jones *et al*,^23^ the mean global score of perceived stigma was 16.54, although again, comparability is hardly possible due to the varying methodology. Future studies using the anticipated stigma scale presented here should further validate the instrument, also in relation to different medical diagnoses or other potentially stigmatised identities.

### Strengths and weaknesses of the study

Our analyses are based on a large random sample of the German general population. However, of those individuals who were selected and eligible for participation, around 55% were not available or refused to take part in this study. Due to this non-response, a selection bias cannot be ruled out. To mitigate the potential impact on representativeness, we made use of a weighting factor. In applying this, we can ensure that the sample’s sociodemographic profile, e.g., regarding sex or level of education, corresponds to the known distribution in the general German population. An overview of the weighted sociodemographic characteristics is given in von dem Knesebeck *et al*.^25^ Despite these efforts, our results should be interpreted with caution, as we cannot claim representativeness. Moreover, we need to point out that the psychometric analyses were conducted using the unweighted data, as weighting can alter the correlation structure of the items. In stigma research, it is common to use vignettes as a standardised stimulus in surveys conducted among the general public.^18^,^20^ If they are to be used in a telephone survey, they have to be reasonably short and concise. The IBS vignette (please see online supplemental material) has been carefully developed with clinical experts. Nevertheless, it bears the risk of being too short to convey a holistic picture of someone who is living with IBS. This brevity could have potentially impacted our findings, as a more comprehensive vignette might have increased respondents’ empathy and a deeper understanding of the condition, leading to different expressions of anticipated stigma. In addition, we changed the original frequency response format of the scale to a four-point agreement format to accommodate the specifics in CATI (as described in the Methods section). This decision reduces the number of response categories and may reduce variance and affect parametric assumptions. Consequently, interpretability of scale means may differ from studies using the original response format. To further verify our results, we conducted sensitivity analyses. These sensitivity checks supported the one-factor structure reported here (see online supplemental material).

Similar to all interviewer-based surveys, a questionnaire via telephone is prone to produce socially desirable responses and may be even more affected by ‘satisficing’, the fact that answers may not be well thought out, e.g., by ‘speeding’ through the survey^40^ . While social desirability bias is a known issue in stigma research, our study design might help to mitigate this concern. Prior research, such as that by Henderson *et al,*^41^ suggests that interviewer-administered surveys can lead to the underreporting of stigma. However, this primarily relates to stigma directed at others. Since our study focuses on the anticipated stigma of the respondents themselves in a hypothetical situation, we believe that the effect of social desirability is likely to be less pronounced. Nevertheless, we have to keep this in mind when interpreting our results. Furthermore, it should be added that the factor loadings of some items as well as the explained variance (29%) can be considered relatively low. However, this may be the reflection of the fact that it is assessing a complex social-psychological construct. Moreover, the satisfactory internal consistency (Cronbach’s α=0.80) supports the use of the sum score as a reliable indicator of anticipated stigma. Finally, our adapted instrument was developed without direct patient participation. However, we made a conscious effort to retain as much of the content of the original PSS IBS developed with involvement of patients.

### Practical implications

There is only a little research on anticipated stigma in the general population, despite evidence of negative consequences with regard to underutilisation of medical testing or treatment. Results presented here indicate that individuals anticipate that others do not know enough about IBS symptoms, or that the development of symptoms is ascribed to own actions and behaviour.

This study introduced a scale designed to assess anticipated stigma associated with IBS in public surveys. Through the use of vignettes, the scale can potentially be adapted to measure anticipated stigma across different conditions. The proposed scale can be used to quantify stigma levels and identify vulnerable populations. A deeper understanding of anticipated stigma concerning different stigmatised identities can lead to the development of more targeted anti-stigma measures. Drawing on results of the present study, this can for example, be the conveyance of clear facts on the development of IBS symptoms. Further research is needed to verify our results and to test the instrument’s applicability regarding other medical disorders or potentially stigmatised identities.

## Supplementary material

10.1136/bmjopen-2024-097149 online supplemental file 1

## Data Availability

Data are available upon reasonable request.
