# Supplementary material for: Measuring anticipated stigma towards irritable bowel syndrome (IBS) in the German general population: testing the applicability of a modified version of the Perceived Stigma Scale of IBS in the cross-sectional SOMA.SOC study
Source: BMJ Open. 2025 Nov 4;15(11):e097149. doi: 10.1136/bmjopen-2024-097149 (PMC12587962; doi:10.1136/bmjopen-2024-097149)
Supplement: online supplemental file 1 [file bmjopen-15-11-s001.docx]

**Supplementary Material
IBS vignettes used in the study**

Ten years ago, 37-year-old Gülsen E. came to Germany from Turkey and she works as a cleaner.^1^ For many months, she has been suffering from frequently recurring abdominal cramps, flatulence and diarrhoea. These troubles put a great strain on Ms. E.'s everyday life and her quality of life is severely restricted as a result. Ms. E. tells her doctor that she has not lost any weight, has no fever or blood in her stool. Examinations up to now have not provided any indication of a threatening disease.

37-year-old Martin E. works as a lawyer.^1^ For many months, he has been suffering from frequently recurring abdominal cramps, flatulence and diarrhoea. These troubles put a great strain on Mr. E.'s everyday life and his quality of life is severely restricted as a result. Mr. E. tells his doctor that he has not lost any weight, has no fever or blood in his stool. Examinations up to now have not provided any indication of a threatening disease.

^1^Migration history (yes/no), sex (male/female), and occupational status (lawyer/cleaner) were systematically varied resulting in eight different vignettes. These vignettes were pooled for the analyses in this article.

**Single Items of Anticipated Stigma - German Version**

*Inwieweit finden Sie die folgenden Aussagen zutreffend, wenn Sie selbst von solchen Beschwerden betroffen wären?*

Meine Symptome würden von anderen nicht ernst genommen werden.

Andere würden glauben, dass meine Symptome eher in meinem Kopf sind als körperlich vorhanden.

Ich würde meine Symptome vor anderen verstecken, weil sie mich sonst anders behandeln würden.

Andere würden glauben, dass meine Symptome durch etwas verursacht werden, das ich tue oder getan habe.

Ich hätte nicht das Gefühl, dass ich mit meinen Symptomen so offen sein kann, wie ich gern würde.

Andere wüssten nicht genug über Symptome wie meine.

Andere wären nicht daran interessiert, etwas über meine Symptome zu hören.

Wenn andere von meinen Symptomen wüssten, würden sie mich anders behandeln.

Andere würden nicht verstehen, wenn ich aufgrund meiner Symptome Pläne ändern müsste.

Ich würde mir Sorgen machen, dass andere mich übergehen oder mich in meinen Möglichkeiten einschränken würden, wenn sie von meinen Symptomen wüssten.

**Results and Interpretation Sensitivity Analyses**

An exploratory factor analysis (EFA) was conducted using Principal Axis Factoring to examine the factor structure of the scale. Given the ordinal nature of the items, a polychoric correlation matrix was used for the analysis. A one-factor solution was specified, with the results indicating that a single factor adequately accounted for the relationships among the ten items.

The factor loadings for the one-factor model are presented in Table X1. All items loaded substantially onto the single factor, with standardized loadings ranging from 0.39 to 0.72. The total variance explained by the single factor was 34%.

**Table X1** Standardized loadings based upon correlation matrix

| **Item** | **Stand. Factor Loadings** | **Communalities** |
| --- | --- | --- |
| 1. My symptoms would not be taken seriously by other people. | 0.57 | 0.32 |
| 2. My symptoms would be believed to be more ‘in my head’ than physical. | 0.56 | 0.31 |
| 3. I would keep my symptoms hidden from other people because they would treat me differently. | 0.64 | 0.41 |
| 4. My symptoms would be believed to be caused by something I’m doing or have done. | 0.39 | 0.15 |
| 5. I would not feel I could be as open about my symptoms as I’d like to be. | 0.72 | 0.51 |
| 6. Some people would not have enough knowledge about my symptoms. | 0.56 | 0.32 |
| 7. Some people would not be interested in hearing about my symptoms. | 0.59 | 0.35 |
| 8. If some people knew about my symptoms, they would treat me differently. | 0.53 | 0.28 |
| 9. Some people would not understand if I had to make changes to plans because of my symptoms. | 0.56 | 0.32 |
| 10. I would worry that some people would pass me over or limit my opportunities if they knew I had these symptoms. | 0.65 | 0.43 |

The fit of the model was evaluated using several key indices. The Tucker-Lewis Index (TLI) of 0.84 indicates a moderate fit (Hu & Bentler, 1999). The Root Mean Square Error of Approximation (RMSEA) was 0.10 (90% CI: 0.09–0.11), which is above the conventional threshold for good fit (Hu & Bentler, 1999). The root mean square of the residuals (RMSR) was 0.06, indicating that the model's residuals were relatively small (Byrne, 1994). Finally, the empirical chi-square value was statistically significant, χ2(35) = 396.39, p < 0.001, but this test is highly sensitive to sample size. Overall, the results support a one-factor structure for the scale, with all items contributing meaningfully to the single latent construct.

References
Byrne, B. M. (1994). Structural equation modeling with EQS and EQS/Windows. Thousand Oaks, CA: Sage Publications.

Hu, L. T., & Bentler, P. M. (1999). Cutoff criteria for fit indexes in covariance structure analysis: Conventional criteria versus new alternatives. Structural Equation Modeling: A Multidisciplinary Journal, 6(1), 1-55.
