# Supplementary material for: Measuring anticipated stigma towards irritable bowel syndrome (IBS) in the German general population: testing the applicability of a modified version of the Perceived Stigma Scale of IBS in the cross-sectional SOMA.SOC study
Source: BMJ Open. 2025 Nov 4;15(11):e097149. doi: 10.1136/bmjopen-2024-097149 (PMC12587962; doi:10.1136/bmjopen-2024-097149)
Supplement: Supplementary file 2 [file bmjopen-2024-097149.reviewer_comments.pdf]

PEER REVIEW HISTORY

BMJ Open publishes all reviews undertaken for accepted manuscripts. Reviewers are asked to complete a checklist review form and are provided with free text boxes to elaborate on their assessment. These free text comments are reproduced below.

ARTICLE DETAILS

Title (Provisional)

Measuring anticipated stigma toward irritable bowel syndrome (IBS) in the German general population: Testing the applicability of a modified version of the Perceived Stigma Scale of IBS in the cross-sectional SOMA.SOC study

Authors

Makowski, Anna Christin; Barbek, Rieke; Toussaint, Anne; Löwe, Bernd; von dem Knesebeck, Olaf

---

VERSION 1 - REVIEW

Reviewer 1

Name Schindler, Stephanie

Affiliation University of Leipzig Medical Center, Department of Psychiatry and Psychotherapy, Leipzig, Germany

Date 06-Jun-2025

COI None

-----------------Article summary -----------------------

*****The peculiar combination of title “Summary” and subtitle “Strengths and Limitations” (set by the journal, I expect?) could be somewhat reconciled if you contrasted your major findings with the corresponding strengths and limitations, e.g., “We report acceptable psychometric properties of a new measure but cannot rule out selection bias in the underlying estimation sample due to a high non-responder rate”.

*****The third point of the list ends with a single word, opening a sentence. *****The fourth point cannot be understood without reading the discussion or without further contextualization (there would be less need for abstraction if you had a sample of affected people).

--------------------------Introduction -------------------------

*****I very much enjoyed reading such a convincing motivation for anticipated stigma research. When you go into the details of existing instruments, it gets a little bit confusing though. In your estimation, Quinn & Chaudoir or the CIASS lack exactly what capacity? It

sounds like you found them not to be specific enough for IBS, but then your own scale, the reader later realizes, also uses general wording. Maybe you could summarize why you preferred adapting Jones's scale to anticipated stigma, plus erasing its specificity to IBS over translating one of the other two scales (which already used general wording and targeted anticipated stigma).

*****On page 6, line 17, you could be more specific, the general population of North Korea might differ from that of the USA – we hope (;

--------------------------Method -----------------------------------

*****On page 8, line 41-45, the sequence of sentences implies that your colleague reports "Another subsample...fatigue" (and not your own). Also, for the reader's convenience please give a short summary of the major sociodemographic characteristics of your sample. A scientific report's main task is to enable the reader to draw her/his own conclusions about the results presented. For this, the reader needs a good idea of the estimation sample and might consider it impolite to be exclusively referred to additional literature to get that.

*****From page 8, line 8, ("in the present study, we solely focus on IBS-related data") onwards, please restrict all vignette/sample/instrument descriptions to the IBS-vignette sample. Please correct me if I'm mistaken, but it seems this should be possible without too many complications and it would greatly reduce the "here and there and back" impression and some redundancies.

*****On pages 10-11, I found the detailed description of the original scale and then the list of your changes intermingled with sample descriptions hard to follow. The reader here wants to know which characteristics of the original scale you deemed worth preserving and which you changed. I got the impression that almost everything was modified, i.e. the raw items' content was kept only, yes? If that is the case, I would have found a short summary of the original psychometric properties of all sixty items (10 items repeated over 6 social domains), along with a short list of your adaptations (e.g., translation, rescaling 5-point scale to 4-point scale, vignette, anticipated stigma, etc.) easier to understand – or at least to keep in mind for interpreting the results.

*****Page 10, line 48: What do you mean by "data was collected from the general German population, so the statements referred to anticipated stigma"? It sounds like asking the general population naturally limits our construct space to this one construct?

*****Page 11, line 23: I think it's quite common to list the initials of the involved members.

*****Page 11, line 39: In scientific contexts, the term "residual" is strongly framed by its statistical meaning, maybe you might want to use another word for the "don't know" answer category.

*****Page 11, line 43: The reader will be very much interested in your reasons for reducing a 5-point scale (consensually still accepted as continuous) to a 4-point scale (ordinal data level; non-parametric statistics needed). Please also include a reflection on the benefits and

downsides of this decision in the discussion – in how far does this limit the interpretability of your results? The “don’t know” answer category will have cost quite some power (N) I expect? Item difficulties, as arithmetic means, require continuous data and normality. Factor analyses require multivariate normality, so even the original 5-point scaling of the items would have stretched this assumption to its limits (if at least equidistant with symmetric distribution and robust estimators and large N). Could you have used a PCA instead?

*****Page 11, line 55: Please also add a nonparametric estimate of variability (e.g., IQR).

*****Page 12, line 8: Bortz & Döring’s recent edition is from 2016. Also, it appears you adjusted their formula for item difficulty to your item scaling, but your formula cannot be understood without definitions of the symbols.

*****Page 12, line 12: Please confirm the wording “the scale was identified using EFA”. Identification usually refers to the uniqueness of solutions for statistical model parameters.

*****Page 12, line 39: “between 1.0 (…) and 4.7% (…).” Here’s a % missing (:

*****Page 12, line 43: “In terms of item difficulty, it was shown that…” This sounds like a citation, but no references are given. If these are your own results, you could indicate this using “we found”.

*****Page 13, line 22: Reporting the mean and SD of the sum score in words here seems sufficient. The corresponding row in Table 3 seems inconsistent with the overall table style and the entries are the same (redundancy in APA terms).

*****Page 13, line 23: You summarize that the histograms and Q-Q-plots “suggested a normal distribution of the data” (sum score?). This section seriously needs clarification. I very rarely encounter a stigma measure that fulfills parametric assumptions — yet not so rarely do I meet colleaques looking for the easiest way out (; As your description could tempt the ill-disposed reader to shortcut testing their own data acquired with your measure and instead just citing your words in order to reduce analysis efforts, I entreat you to very strictly test the normality and show the results (skewness, kurtosis, Q-Q-plot, and density distribution with normality curve overlay) to substantiate your impression.

----------------------Discussion -------------------------

*****29% variance explained by a single-factor solution is rather low in my experience. What does it mean for the interpretation of the sum score and the psychometric value of the instrument? Does it mean that roughly two thirds of the variance of the sum score (unweighted and hence assuming a one-dimensional construct) is essentially noise?

*****I’d have loved a short reflection on the fact that the scale’s adaptation happened purely non-participatory. Maybe people experiencing IBS were involved in the development of the original scale?

--------------------Miscellanea -------------------

*****Did you use any AI during the analysis or writing process? If so, please specify in accordance with the journal's or APA citation guidelines.

*****Please note: ChatGPT kindly assisted with language editing of this review (:

**Reviewer**	**2**
**Name**	**Dharmaraj, Rajmohan**
**Affiliation**	**University of New Mexico, Rajmohan Dharmaraj**
**Date**	**17-Aug-2025**
**COI**	**None**

Anna Makowski et al. examined anticipated stigma toward irritable bowel syndrome (IBS) in the general population, using a modified Perceived Stigma Scale of IBS. As part of the SOMA.SOC project, 1,205 German adults participated in telephone interviews featuring IBS symptom vignettes. The study tested the scale's applicability and psychometric properties. Results indicated participants expected stigma, such as others lacking IBS knowledge or attributing symptoms to behavior. A single-factor solution with strong internal consistency (Cronbach's $\alpha = 0.80$) emerged. Findings showed moderate anticipated stigma, suggesting the scale's utility across conditions. The study highlights the need to address stigma to improve care and quality of life for those with IBS and recommends further validation of the scale.

This study has several strengths. The topic is highly relevant to clinical practice. It is based on a large, randomly selected sample of the German general population, increasing its representativeness. The use of carefully developed vignettes, created in collaboration with clinical experts, provides a standardized and engaging method to assess complex medical conditions like IBS. The study also introduces a modified stigma scale tailored for population-level surveys, demonstrating good psychometric reliability (Cronbach's $\alpha = 0.80$). Additionally, the research fills a gap in stigma studies by focusing on anticipated stigma rather than perceived stigma, offering valuable insights for developing targeted anti-stigma interventions. The findings can help improve healthcare utilization and awareness.

To enhance the manuscript, consider addressing these minor issues: discuss strategies to mitigate the impact of the 55% non-response rate, which may affect representativeness; explore measures to reduce socially desirable responses; expand the vignettes to provide a more comprehensive depiction of IBS, which could increase respondents' empathy and understanding; and involve patients in the design process to improve relevance and accuracy.

VERSION 1 - AUTHOR RESPONSE

Reviewer: 1
Dr. Stephanie Schindler, University of Leipzig Medical Center, Department of Psychiatry and Psychotherapy, Leipzig, Germany
Comments to the Author:

Author´s response: Thank you very much for your valuable feedback. Please find a response to the points you have raised in the following.

-----------------Article summary -----------------------

*****The peculiar combination of title “Summary” and subtitle “Strengths and Limitations” (set by the journal, I expect?) could be somewhat reconciled if you contrasted your major findings with the corresponding strengths and limitations, e.g., “We report acceptable psychometric properties of a new measure but cannot rule out selection bias in the underlying estimation sample due to a high non-responder rate”.

- We appreciate your comment. We followed the journal’s guideline here. It is recommended that this section should be relating specifically to the methods - not the results of the study. However, we agree that we can strengthen these points and have therefore decided on the following rephrasing (p 3), in the hope that it will still align with the journal's requirements:

    - *We report acceptable psychometric properties of a modified Perceived Stigma Scale for IBS in a large random sample of the German general population.*
    - *Case vignettes developed by clinicians and specialists allowed for the presentation of the complex medical condition of IBS in a standardized way.*
    - *A non-response rate of 55% raises the possibility of selection bias.*
    - *The assessment of anticipated stigma in the general population required respondents to abstract from a vignette rather than drawing on personal illness experience.*

*****The third point of the list ends with a single word, opening a sentence.

- This has been deleted, thanks for pointing that out.

*****The fourth point cannot be understood without reading the discussion or without further contextualization (there would be less need for abstraction if you had a sample of affected people).

- We agree with you on this point and have rephrased the sentence accordingly. It now reads as follows (p 3):
  “*The assessment of anticipated stigma among the general public was primarily conducted with participants who do not have IBS, requiring them to abstract from a vignette instead of relying on own illness experiences.”*

-------------------------Introduction -------------------------

*****I very much enjoyed reading such a convincing motivation for anticipated stigma research. When you go into the details of existing instruments, it gets a little bit confusing though. In your estimation, Quinn & Chaudoir or the CIASS lack exactly what capacity? It sounds like you found them not to be specific enough for IBS, but then your own scale, the reader later realizes, also uses general wording. Maybe you could summarize why you preferred adapting Jones’s scale to anticipated stigma, plus erasing its specificity to IBS over translating one of the other two scales (which already

used general wording and targeted anticipated stigma).

Thank you for your comment. We appreciate the opportunity to clarify our choice of the scale.

Our preference for the Jones et al. scale was driven by its conceptual fit with the project's primary focus on IBS, as it was developed specifically for this condition. However, given our methodological choice to use vignettes, and our broader goal of assessing a second condition, fatigue, we saw an opportunity to adapt the scale. This decision allowed us to maintain the benefits of a concrete, situation-based conceptual framework. In contrast to the even more abstract and general scales like the CIASS or the one by Quinn and Chaudoir, we found the items of the adapted PSS IBS a better fit for capturing direct, context-related reactions to the specific cases presented in the vignettes. By using a single, adapted instrument, we wanted to ensure methodological consistency and allow for the comparability of anticipated stigma responses across both conditions.

We have also revised our Introduction section accordingly, hoping to be clearer now. Please find the following paragraph on p 6:

"*While there are scales to measure anticipated stigma, we adapted the condition-specific scale developed by Jones et al. [23], originally designed for IBS. Our rationale was that the IBS context is unique, so starting from an IBS-specific instrument was preferable. The aforementioned instruments [7, 16] were broader, but not ideal for vignette-based population survey research focused on IBS. For this purpose, we used a modified version of the PSS IBS. This study aimed to test the modified scale with regard to its applicability and psychometric properties.*"

*****On page 6, line 17, you could be more specific, the general population of North Korea might differ from that of the USA – we hope (;

- We're sorry if we don't understand this comment correctly, or if we've gotten a line wrong. However, both of the cited studies were conducted in the U.S.

--------------------------Method ------------------------------------

*****On page 8, line 41-45, the sequence of sentences implies that your colleague reports "Another subsample...fatigue" (and not your own).

- Thank you for your comments regarding this aspect in the methods section. We certainly agree with you. Following your suggestion, we revised the text (now on p 7) to clarify the sentence sequence. The text now explicitly states that both the IBS and fatigue subsamples are part of our overall study, eliminating any potential confusion about their origin:

"At the beginning of the interviews, a vignette describing a person with typical symptoms of IBS or fatigue was presented to the respondents (for details please see below and Supplementary Material). One of the two vignettes (IBS or fatigue) was randomly assigned to

half of the sample respectively. As the following analyses will focus on IBS, half of the total sample (n = 1,205) will be used."

Also, for the reader's convenience please give a short summary of the major sociodemographic characteristics of your sample. A scientific report's main task is to enable the reader to draw her/his own conclusions about the results presented. For this, the reader needs a good idea of the estimation sample and might consider it impolite to be exclusively referred to additional literature to get that.

- Regarding a brief description of the sample size (as nothing could be further from our intention than to appear impolite), we are happy to now provide a few more details (p 7):

  "*The overall study sample was comprised of 49.5% female respondents, with an average age of 52 years (SD = 18.5). Around 37% had high level of education (≥ 12 years). For more details, please also see the publication by von dem Knesebeck et al. [25].*"

*****From page 8, line 8, ("in the present study, we solely focus on IBS-related data") onwards, please restrict all vignette/sample/instrument descriptions to the IBS-vignette sample. Please correct me if I'm mistaken, but it seems this should be possible without too many complications and it would greatly reduce the "here and there and back" impression and some redundancies.

- You are absolutely right. We have revised the corresponding text section so that it now exclusively refers to the IBS vignette. This change is probably most evident in the "Vignettes" sub-section of the Methods section on page 8.

*****On pages 10-11, I found the detailed description of the original scale and then the list of your changes intermingled with sample descriptions hard to follow. The reader here wants to know which characteristics of the original scale you deemed worth preserving and which you changed. I got the impression that almost everything was modified, i.e. the raw items' content was kept only, yes? If that is the case, I would have found a short summary of the original psychometric properties of all sixty items (10 items repeated over 6 social domains), along with a short list of your adaptations (e.g., translation, rescaling 5-point scale to 4-point scale, vignette, anticipated stigma, etc.) easier to understand – or at least to keep in mind for interpreting the results.

- We agree with you and have made an effort to rearrange the subchapter "Instrument" of the Methods section you are referring to. Now, we first summarize the original scale's properties and then describe our specific adaptions in a second section.
  Given the extensive changes made to this sub-section, we have decided not to include them in detail in the point-by-point response. Instead, we would like to refer you to pages 9 and 10 of the revised manuscript and hope that we have successfully addressed your comments.

*****Page 10, line 48: What do you mean by "data was collected from the general German population, so the statements referred to anticipated stigma"? It sounds like asking the general population naturally limits our construct space to this one construct?

- Thank you for your comment. We did not intend to imply that surveying the general population automatically limits the construct to anticipated stigma. A general population sample could indeed capture a variety of constructs. The point we wanted to bring across was that our methodological design (involving vignettes and using the modified version of the PSS IBS) was tailored to measure anticipated stigma. As stated in our introduction, this is a construct that, unlike perceived or experienced stigma, is not contingent on direct personal experience. It can also be present in the general population because it reflects

hypothetical views on how individuals with a potentially stigmatized condition might be assessed and treated.
To be clearer, we have rephrased the sentence in the section Adaptions for the current study (p 10):

"*Whereas the PSS IBS measures perceived stigma, our study used vignettes and surveyed the general population, a methodological approach specifically designed to measure anticipated stigma in individuals both with and without lived experience of the condition, as this type of stigma reflects hypothetical views and is not contingent on direct personal experience.*"

*****Page 11, line 23: I think it's quite common to list the initials of the involved members.

- We agree with you and have added this information accordingly.

*****Page 11, line 39: In scientific contexts, the term "residual" is strongly framed by its statistical meaning, maybe you might want to use another word for the "don't know" answer category.

- Yes, we have decided to rephrase the sentence as follows (p 11):
  "*Respondents who could not or did not want to commit themselves when answering the questions were given the option of selecting a "don't know" / "no answer" category.*"

*****Page 11, line 43: The reader will be very much interested in your reasons for reducing a 5-point scale (consensually still accepted as continuous) to a 4-point scale (ordinal data level; non-parametric statistics needed). Please also include a reflection on the benefits and downsides of this decision in the discussion – in how far does this limit the interpretability of your results? The "don't know" answer category will have cost quite some power (N) I expect?

- Thank you for your detailed comments on our methodological choices. We agree that the decision to use a 4-point scale can be seen as a critical one. In their original paper, Jones et al. (2009) report a scale from 0 to 5, which corresponds to six points. However, only the endpoints were verbalized, which is difficult to implement in a telephone survey. Later in their work, they refer to a 5-point scale without providing further details. Subsequent studies, including those in the context of inflammatory bowel disease, have also used a 5-point version of the scale. As our study asks participants to respond to hypothetical situations—which we are aware requires a certain level of abstraction—we wanted to avoid a central tendency bias and instead offer a 'don't know' category. We have added a short justification in the Methods section, explaining the 4-point agreement format (p 10):

  "*To suit the context of our study, we modified the original scale's response options. This design decision facilitated clear communication during the telephone interviews, reduced respondent burden, and avoid a tendency to the middle. A fully verbalized four-point response scale was used ("completely disagree", "rather disagree", "rather agree", "completely agree"), thus measuring the degree of agreement with the individual items.*"

- Moreover, we now report the performance of sensitivity analyses (also in the Methods section, Statistical Analyses, p 13):

  "*In addition, given the ordinal nature of the item response, we performed sensitivity analyses. A polychoric correlation matrix was calculated using the psych package in R. EFA*

was then performed on this matrix using principal axis factoring to extract a single factor. These analyses were conducted using the fa.poly function from the psych package in R [34]. For results of sensitivity analyses, please see Supplementary Material."

To avoid further extending the already extensive manuscript, we have decided to include the results in the supplementary material. Please also see it attached to this point-by-point response.
We are aware that the reduction to a 4-point scale can be seen critical. Nevertheless, we hope we have adequately addressed this point and have also added a limitations paragraph in the Discussion (p 17):

"*In addition, we changed the original frequency response format of the scale to a four-point agreement format to accommodate for the specifics in CATI (as described in the Methods section). This decision reduces the number of response categories and may reduce variance and affect parametric assumptions. Consequently, interpretability of scale means may differ from studies using the original response format. To further verify our results, we conducted sensitivity analyses. These sensitivity checks supported the one-factor structure reported here (see Supplementary Material).*"

- Pertaining to your comment regarding missing values:
  We agree with you that any missing data, including "don't know" responses, can technically reduce statistical power. However, to address cases where participants genuinely could not form a directional opinion, we wanted to include the separate "don't know" option. For our study on anticipated stigma, we deemed this particularly important, as we wanted to allow participants to honestly admit if they couldn't project themselves into the hypothetical situation. By allowing participants to say "I don't know," we wanted to avoid forcing them to give a speculative answer that could also introduce a source of bias:
  We regarded the amount of missings (between 1% and 4.7%, please also see results section, p 13) as still relatively low and thereby acceptable.

Item difficulties, as arithmetic means, require continuous data and normality. Factor analyses require multivariate normality, so even the original 5-point scaling of the items would have stretched this assumption to its limits (if at least equidistant with symmetric distribution and robust estimators and large N). Could you have used a PCA instead?

- We agree that the chosen EFA with maximum likelihood assumes multivariate normality. To address this, we now report skewness/kurtosis for items and the sumscore, and ran sensitivity analyses. The alternative methods produced substantively the same one-factor solution; these results are reported in the Supplementary Material. Moreover, given the considerably large sample size, we consider ML estimation as relatively robust.

- Pertaining to your suggestion of using principal component analysis instead, we would like to explain our choice of exploratory factor analysis: We used EFA rather than PCA because the aim was to model a latent construct (anticipated stigma) rather than simply reduce variables. Since PCA is a variance-maximization technique and does not model latent constructs, we considered EFA more appropriate for our rationale of scale development and validation.

*****Page 11, line 55: Please also add a nonparametric estimate of variability (e.g., IQR).

- We have now added IQR on p 12 as well as in an additional column in table 2.

*****Page 12, line 8: Bortz & Döring's recent edition is from 2016. Also, it appears you adjusted their formula for item difficulty to your item scaling, but your formula cannot be understood without definitions of the symbols.

- Pertaining to the publication of Bortz and Döring, we'd like to thank you for pointing this out. There's even a 6th edition from 2023 now. We have updated our reference list!
- We agree with you that the presentation of the formula is not very intuitive. Instead, we now just describe the computation of item difficulty as follows (p 12):

  "*We calculated the item difficulty by dividing the item mean by the maximum possible response category (i.e., 3).*"

*****Page 12, line 12: Please confirm the wording "the scale was identified using EFA". Identification usually refers to the uniqueness of solutions for statistical model parameters.

- We agree with you regarding this, thanks for pointing it out. Instead of "identified" we now speak of "*explored*".

*****Page 12, line 39: "between 1.0 (…) and 4.7% (…)." Here's a % missing (:

- Thank you for pointing this out, all % are present now.

*****Page 12, line 43: "In terms of item difficulty, it was shown that…" This sounds like a citation, but no references are given. If these are your own results, you could indicate this using "we found".

- Upon your suggestion we have rephrased this sentence in the Results sections which now reads as follows (p 13):
  "In terms of item difficulty, we found that the item "Some people would not have enough knowledge..." had the lowest difficulty (0.59), while the highest item difficulty value was achieved for the item "I would keep my symptoms hidden...." (0.38)."

*****Page 13, line 22: Reporting the mean and SD of the sum score in words here seems sufficient. The corresponding row in Table 3 seems inconsistent with the overall table style and the entries are the same (redundancy in APA terms).

- You are correct in pointing that out. To avoid redundancies, and also in order address the inconsistent table style, we have deleted the row presenting mean and SD of the sum score from table 3.

*****Page 13, line 23: You summarize that the histograms and Q-Q-plots "suggested a normal distribution of the data" (sum score?). This section seriously needs clarification. I very rarely encounter a stigma measure that fulfills parametric assumptions — yet not so rarely do I meet colleaques looking for the easiest way out (; As your description could tempt the ill-disposed reader to shortcut testing their own data acquired with your measure and instead just citing your words in order to reduce analysis efforts, I entreat you to very strictly test the normality and show the results (skewness, kurtosis, Q-Q-plot, and density distribution with normality curve overlay) to substantiate your impression.

- We appreciate your comment and are happy to address this point in more detail.
  First, to provide greater transparency, we've now added skewness, kurtosis, and their corresponding standard errors to the results section (p 14):

  "*The stigma sum score was approximately normally distributed, with a mean of 14.54 (SD = 5.46) and a median of 15.00. The data exhibited a skewness of -0.062 (SE = 0.075) and a*

kurtosis of -0.121 (SE = 0.149), with histograms and Q-Q plots further supporting a normal distribution."

Based on the histogram and Q-Q plot, we would also like to continue to refer to an approximate normal distribution. Given the current length of the manuscript, we've decided not to include the graphs in the main text. However, we are happy to provide them here in our response, and hope this adequately addresses your concern.

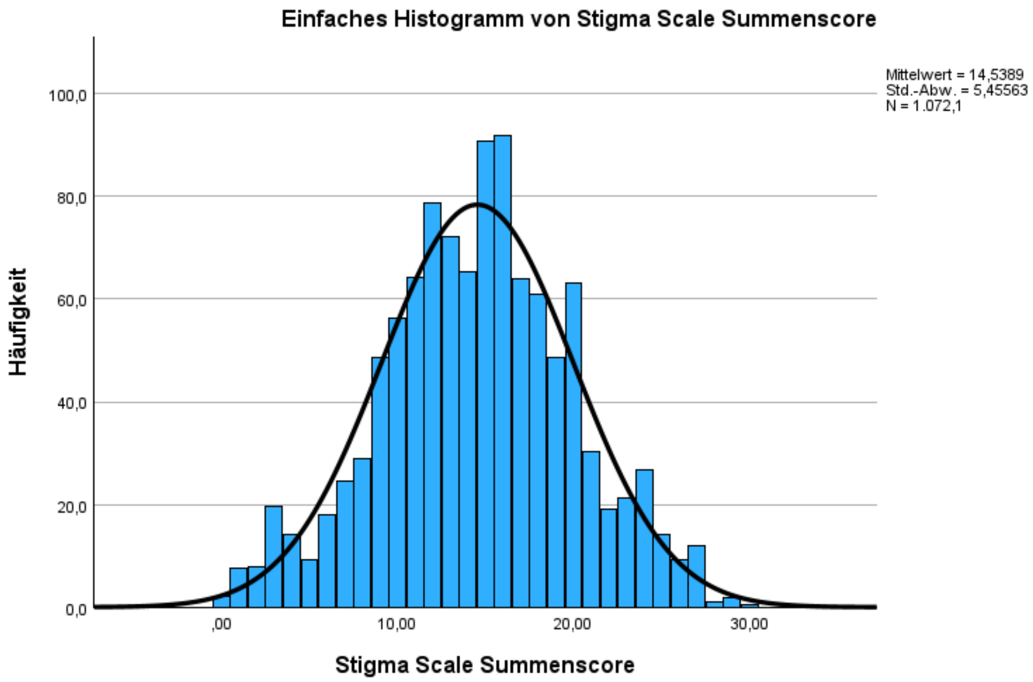

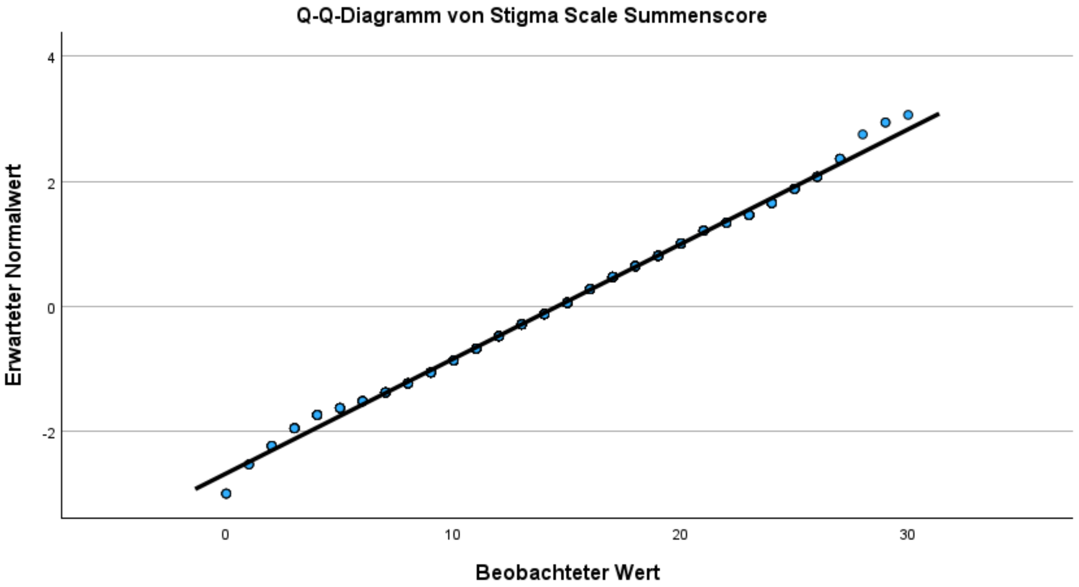

----------------------Discussion --------------------------

*****29% variance explained by a single-factor solution is rather low in my experience. What does it mean for the interpretation of the sum score and the psychometric value of the instrument? Does it mean that roughly two thirds of the variance of the sum score (unweighted and hence assuming a one-dimensional construct) is essentially noise?

- We agree with you that the low percentage of variance explained requires some more attention. However, we wouldn't regard it necessarily just as "noise" in the sense of random error. It may be a reflection of the fact that it is assessing a complex social-psychological construct. Moreover, the satisfactory internal consistency (Cronbach's α = 0.80) supports the use of the sum score as a reliable indicator of anticipated stigma. The unexplained variance likely reflects the multifaceted nature of stigma and individual differences in response patterns, rather than pure measurement noise.
  We appreciate you bringing this aspect to our attention, as it highlights that we have to discuss it clearer and more concise as a limitation. We have added the following paragraph to the limitations section on page 17, hoping that it is adequately addressing your concerns:

  "*Furthermore, it should be added that the factor loadings of some items as well as the explained variance (29%) can be considered relatively low. However, this may be the reflection of the fact that it is assessing a complex social-psychological construct. Moreover, the satisfactory internal consistency (Cronbach's α = 0.80) supports the use of the sum score as a reliable indicator of anticipated stigma.*"

*****I'd have loved a short reflection on the fact that the scale's adaptation happened purely non-participatory. Maybe people experiencing IBS were involved in the development of the original scale?

- Thank you for this comment. You are correct; the development of our adapted scale was non-participatory. However, we would like to confirm that the original PSS-IBS by Jones et al. (2009) was developed with the direct involvement of patients through structured interviews. This initial, patient-centered approach ensured the scale's items were conceptually grounded in the lived experiences of individuals with IBS. The final 10-item scale was then validated through a survey with an additional 148 IBS patients.
  While our adaptation was non-participatory, we made a conscious effort to stay conceptually close to the original scale to retain its patient-relevant content. However, we were not able to cover all six social domains / contexts (e.g., family, work, health care provider, etc.). These aspects represent a limitation of our study, which we have now included in the limitations section of the manuscript (p 17):

  "*Finally, our adapted instrument was developed without direct patient participation. However, we made a conscious effort to retain as much of the content of the original PSS IBS developed with involvement of patients.*"

--------------------Miscellanea -------------------

*****Did you use any AI during the analysis or writing process? If so, please specify in accordance with the journal's or APA citation guidelines.

- No AI was used during the initial analysis or writing process. However, in writing this point-by-point response, I relied on gemini's language editing in some cases.

******Please note: ChatGPT kindly assisted with language editing of this review (:
